# ^68^Ga-PSMA-11 PET/CT Features Extracted from Different Radiomic Zones Predict Response to Androgen Deprivation Therapy in Patients with Advanced Prostate Cancer

**DOI:** 10.3390/cancers14194838

**Published:** 2022-10-03

**Authors:** Vuong Thuy Tran, Shu-Ju Tu, Jing-Ren Tseng

**Affiliations:** 1 Department of Medical Imaging and Radiological Sciences, College of Medicine, Chang Gung University, Tao-Yuan 333, Taiwan; 2 Department of Medical Imaging and Intervention, Linkou Chang Gung Memorial Hospital, Tao-Yuan 333, Taiwan; 3 Department of Nuclear Medicine, Linkou Chang Gung Memorial Hospital, Tao-Yuan 333, Taiwan; 4 Department of Nuclear Medicine, New Taipei Municipal TuCheng Hospital (Built and Operated by Chang Gung Medical Foundation), New Taipei City 236, Taiwan; 5 School of Medicine, Chang Gung University, Tao-Yuan 333, Taiwan

**Keywords:** radiomics, prostate cancer, ^68^Ga-PSMA-11 PET, radiomic zones, androgen deprivation therapy, treatment response

## Abstract

**Simple Summary:**

Androgen deprivation therapy plays a key role in the therapeutic management of patients with advanced prostate cancer. However, prediction of response before treatment initiation remains difficult. This study was undertaken to investigate whether ^68^Ga-PSMA-11 PET/CT imaging features extracted from different prostatic zones (zone-1, zone-2, and zone-3) might predict response to androgen deprivation therapy in patients with advanced prostate cancer. Seven radiomic features extracted from zone-1 were identified as significantly associated with treatment response. In addition, two radiomic features from zone-2 and two from zone-3 were able to distinguish between different treatment response groups. Our findings demonstrate the potential usefulness of radiomic features extracted from different prostatic zones in predicting treatment response prior to androgen deprivation therapy.

**Abstract:**

**Purpose:** Prediction of treatment response to androgen deprivation therapy (ADT) prior to treatment initiation remains difficult. This study was undertaken to investigate whether ^68^Ga-PSMA-11 PET/CT features extracted from different radiomic zones within the prostate gland might predict response to ADT in patients with advanced prostate cancer (PCa). **Methods:** A total of 35 patients with prostate adenocarcinoma underwent two ^68^Ga-PSMA-11 PET/CT scans—termed PET-1 and PET-2—before and after 3 months of ADT, respectively. The prostate was divided into three radiomic zones, with zone-1 being the metabolic tumor zone, zone-2 the proximal peripheral tumor zone, and zone-3 the extended peripheral tumor zone. Patients in the response group were those who showed a reduction ratio > 30% for PET-derived parameters measured at PET-1 and PET-2. The remaining patients were classified as non-responders. **Results:** Seven features (glcm_idmn, glcm_idn, glcm_imc1, ngtdm_Contrast, glrlm_rln, gldm_dn, and shape_MeshVolume) from zone-1, two features (gldm_sdlgle and shape_MinorAxisLength) from zone-2, and two features (diagnostics_Mask-interpolated_Minimum and shape_Sphericity) from zone-3 successfully distinguished responders from non-responders to ADT. One predictive feature (shape_SurfaceVolumeRatio) was consistently identified in all of the three zones. **Conclusions:** this study demonstrates the potential usefulness of radiomic features extracted from different prostatic zones in distinguishing responders from non-responders prior to ADT initiation.

## 1. Introduction

Globally, prostate cancer (PCa) is the second most common cancer among men and the fifth leading cause of death [1]. The role of positron emission tomography (PET) imaging in the diagnosis, staging, and assessment of treatment response in patients with PCa is well-established [2]. ^68^Ga-labeled prostate-specific membrane antigen (PSMA)-targeted PET has recently emerged as a promising imaging modality for lesion detection [3,4] and monitoring of response to androgen deprivation therapy (ADT) in patients with metastatic PCa [5,6]. There is also evidence that the percentage variations of ^68^Ga-PSMA-11 PET/CT imaging parameters measured before and after three months of ADT are clinically useful in evaluating treatment response [7,8,9].

PCa is characterized by significant intratumor heterogeneity, which may in turn affect the biological aggressiveness, disease progression, and therapeutic resistance [10,11,12]. In recent years, radiomics—which can be defined as the high-throughput extraction of quantitative features from CT, MRI, or PET images—has been successfully used to predict clinical and treatment outcomes in patients with malignancies [13,14,15,16,17,18,19,20,21]. Image texture analysis—as a quantitative radiomics approach for the analysis of tumor heterogeneity—has been shown to correlate with established indices of glucose metabolic activity, including the standardized uptake value (SUV) [22,23,24,25]. Moreover, texture features may have a complementary role to metabolic parameters in the prediction of treatment response [26].

The ability of radiomics to comprehensively characterize PCa tissues from state-of-the-art PET imaging has attracted significant research interest [27,28,29,30,31,32]. On analyzing ^18^F-choline PET/CT scans of patients with high-risk PCa, Alongi et al. [30] identified three features (i.e., SUVmin, shape_Sphericity, and idmn_Correlation) that successfully predicted the occurrence of disease progression at follow-up. However, most studies in the field of PET radiomics have continued to rely on a cancer-centric model based on feature extraction from the whole tumor. More recently, the regions of interest have been expanded to include peripheral tumor areas [33,34,35]. In a previous study focusing on ^11^C-choline PET/MRI imaging, we have proposed dividing the prostate gland into three distinct radiomic zones, with zone-1 being the metabolic tumor zone, zone-2 the proximal peripheral tumor zone, and zone-3 the extended peripheral tumor zone [35]. Interestingly, these radiomic zones were found to have different predictive strengths in classifying risk groups in patients with PCa [35].

While measurements of circulating prostate specific antigen (PSA) levels may have a role in predicting response to ADT when PCa has not spread to lymph nodes or skeletal sites [36], its clinical utility in patients with disseminated disease remains limited. In this scenario, there is pilot evidence supporting the potential utility of the reduction ratios of ^68^Ga-PSMA-11 PET/CT indices measured before and after 3 months of ADT [7,9]. By expanding our previous work [35], we designed this current study to investigate whether radiomic features from zone-1 may have clinical value for predicting response to ADT. We also examined whether features from peripheral areas (zone-2 and zone-3) could be useful to distinguish between different treatment response groups.

## 2. Materials and Methods

### 2.1. Study Patients

Thirty-five patients with advanced prostatic adenocarcinoma were included (Table 1). All participants were scheduled to undergo ADT for at least 6 months and have completed nearly 3 months (10–14 weeks) of ADT treatment. Two ^68^Ga-PSMA-11 PET/CT scans—termed PET-1 and PET-2—were obtained for each patient before and after 3 months of ADT, respectively. Ethics approval for this study was received from the Chang Gung Memorial Hospital institutional review board (reference number: 201801384A0). All participants provided written informed consent.

### 2.2. PET/CT Imaging

The acquisition protocol for ^68^Ga-PSMA-11 PET/CT imaging has been previously described [9]. In brief, images were acquired on a GE Discovery MI PET/CT scanner (GE Healthcare, Milwaukee, WI, USA) 60 min after tracer injection (dose range: 103–182 MBq; median dose: 141 MBq). The following settings were applied for CT imaging: 120 kVp, automatic mA selection (ranging from 30 to 300 mA), 40 × 0.625 detector collimation, and 0.984 pitch. Transaxial PET images were acquired with the following parameters: field of view = 700 mm, matrix size = 256 × 256, and slice thickness = 5 mm. The acquisition time was 3 min per single-bed position, with the acquisition proceeding from the thigh to the skull. A Bayesian penalized likelihood algorithm (Q.Clear) was used for image reconstruction. Methods for image calibration included attenuation correction, the point spread function, and the QCHD-S technique [9].

### 2.3. PET-Derived Parameters

**Figure 1:**
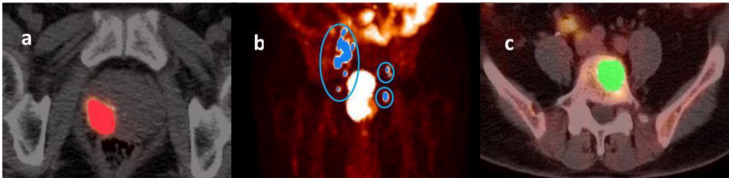
Illustrative images of a primary prostate tumor (panel (**a**)), metastatic lymph nodes (panel (**b**)), and metastatic bone lesions (panel (**c**)) identified on a ^68^Ga-PSMA-11 PET scan performed before the start of androgen deprivation therapy (PET-1). A maximum standardized uptake value (SUV) threshold of 45% was used to delineate the primary prostate tumor (panel (**a**)) and metastatic lymph nodes (panel (**b**)), whereas a fixed-absolute SUV threshold of 3.0 was applied for metastatic skeletal lesions (panel (**c**)).

Region of interest (ROI)-based image segmentation was performed using the LIFEx software developed in Java [37]. A maximum standardized uptake value (SUV) threshold of 45% was used to delineate the primary prostate tumor and metastatic lymph nodes (Figure 1a,b) [38], whereas a fixed-absolute SUV threshold of 3.0 was applied for metastatic bone lesions (Figure 1c) [39].

Traditional PET-derived parameters—including SUVmax, SUVmean, metabolic tumor volume (MTV), and total lesion (TL, calculated by multiplying SUVmean by the MTV)—were used for assessing treatment response [7,9,40]. Calculation of these indices for the primary prostate tumor, metastatic lymph nodes, and bone lesions was implemented on a patient basis using the LIFEx package. All parameters were calculated for both PET-1 and PET-2 images.

### 2.4. Analysis of Treatment Response

Patients who had undergone ADT treatment were classified as either responders or non-responders using the modified PET response criteria in solid tumors (mPERCIST) [41]. With this aim, the primary prostate tumor, metastatic lymph nodes, and bone lesions were taken into account. Patients in the response group were those who showed a reduction ratio (RR) > 30% for PET-derived parameters measured on PET-2 versus PET-1 [41]. The remaining patients were classified as non-responders. The RR was calculated with the following formula:

$$\mathrm{RR}=-\frac{{\mathrm{parameter}}_{\mathrm{PET}\text{-}2}-{\mathrm{parameter}}_{\mathrm{PET}\text{-}1}\;}{{\mathrm{parameter}}_{\mathrm{PET}\text{-}1}}\times 100\%$$

**Figure 2:**
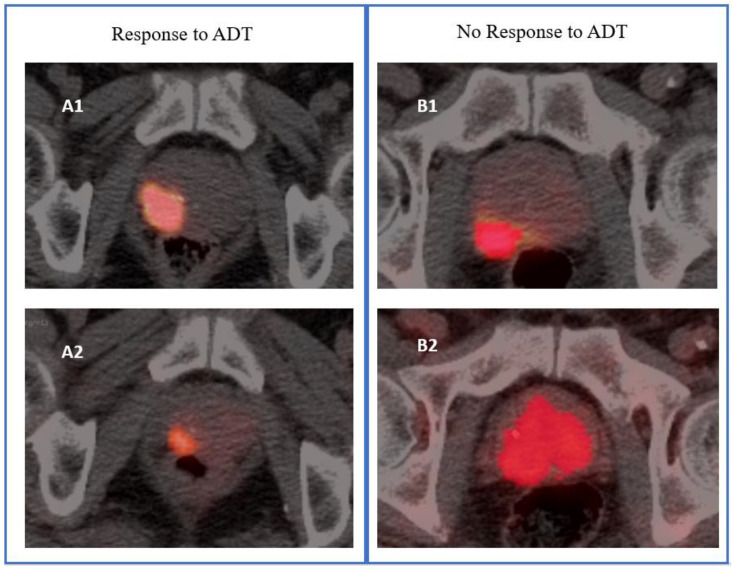
Illustrative images of response and no response to androgen deprivation therapy (ADT) of a primary prostate tumor based on metabolic tumor volume. The (**A1**,**B1**) PET/CT images were from ^68^Ga-PSMA-11 PET scans performed before the start of ADT (PET-1), whereas (**A2**,**B2**) were the corresponding images from ^68^Ga-PSMA-11 PET scans performed after 3 months of ADT (PET-2). Response was defined as a reduction ratio > 30% for PET-derived parameters measured on PET-2 versus PET-1.

Parameters included SUVmax, SUVmean, MTV, or TL, respectively. Illustrative examples of tumor response and non-response are shown in Figure 2.

### 2.5. Radiomic Zones and Feature Extraction

**Figure 3:**
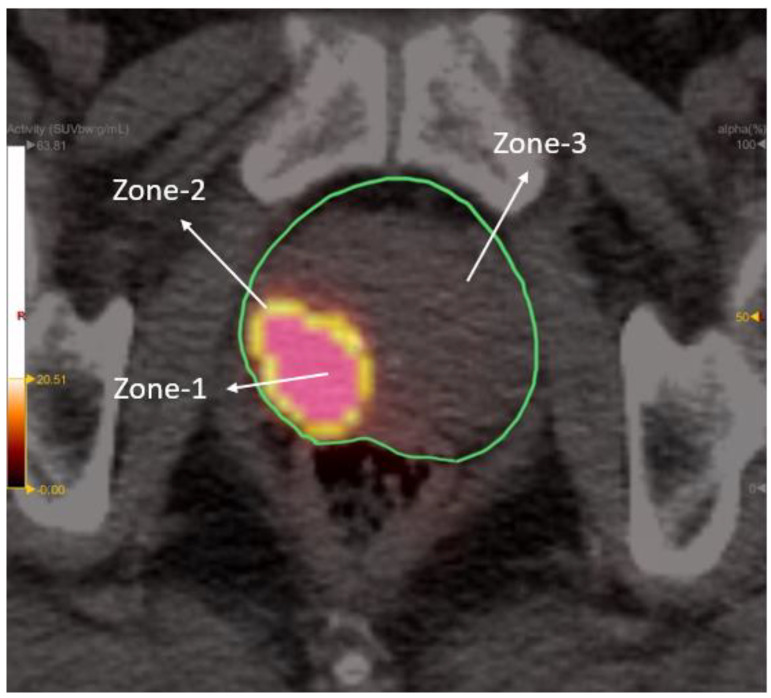
Depiction of the three radiomic zones on a PET/CT image. Standardized uptake values for zone-1 and zone-2 were 45–100% and 20–45% of maximum standard uptake value (SUVmax), respectively. Zone-3 comprised the entire prostate with the exclusion of zone-1.

Radiomic zones (zone-1, zone-2, and zone-3) of the prostate were defined in accordance with our previous study [35]. Specifically, zone-1 is the metabolic tumor region, zone-2 the proximal peripheral region surrounding zone-1, and zone-3 the expanded peripheral region reaching to the prostate boundary. In brief, SUV values for zone-1 and zone-2 were 45–100% and 20–45% of SUVmax, respectively. Zone-3 comprised the entire prostate with the exclusion of zone-1 (Figure 3). After segmentation of the three zones on PET-1 images, radiomics features were extracted through the open-source Python package PyRadiomics [42,43]. A total of 119 PyRadiomics features were examined across the following eight categories: first-order statistics (18 features), diagnosis (12), shape (14), gray level co-occurrence matrix (glcm) (24), gray level dependence matrix (gldm) (14), gray level run length matrix (glrlm) (16), gray level size zone matrix (glszm) (16), and neighboring gray tone difference matrix (ngtdm) (5) [43].

### 2.6. Statistical Analysis

We examined PyRadiomics features (*n* = 119) extracted from the three radiomic zones. Groups with different response to ADT were compared on normally distributed variables using independent Student’s *t*-tests and on skewed parameters with the Mann–Whitney *U* test. In each prostatic zone, a radiomic feature was considered useful when it successfully distinguished ADT response groups and showed an association with treatment outcomes on at least three of the following traditional PET parameters (i.e., SUVmax, SUVmean, MTV, and TL). All analyses were undertaken in SPSS, version 25.0 (IBM, Armonk, NY, USA), and statistical significance was determined by a two-tailed *p* value < 0.05.

## 3. Results

### 3.1. Response to Androgen Deprivation Therapy

Of the 35 patients with PCa who were staged with ^68^Ga-PSMA-11 PET imaging before ADT treatment, 16 had metastatic lymph nodes and 17 had bone metastases. Using the four traditional PET parameters, patients were classified into different treatment response groups (Table 2). On average, the percentage distribution of responders and non-responders for primary tumors (*n* = 35), metastatic lymph nodes (*n* = 16), and bone lesions (*n* = 17) was as follows: 73%/27%, 84%/16%, and 73%/27%, respectively.

### 3.2. Prediction of Treatment Response Using Features from Radiomic Zone-1

**Figure 4:**
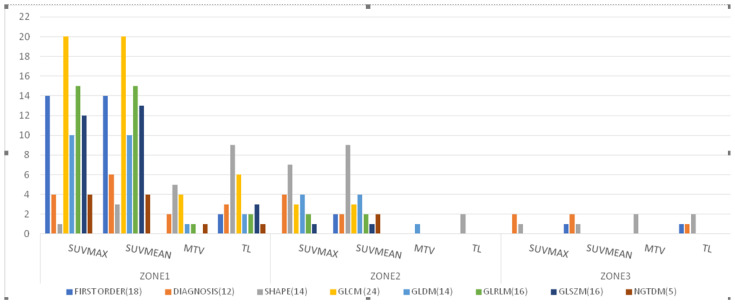
Distribution of features extracted from the three radiomic zones for distinguishing between responders and non-responders to androgen deprivation therapy (*p* < 0.05; independent Student’s *t*-tests or Mann–Whitney *U* test). Features were examined across the following eight categories: first-order statistics (18 features), diagnosis (12), shape (14), gray level co-occurrence matrix (glcm) (24), gray level dependence matrix (gldm) (14), gray level run length matrix (glrlm) (16), gray level size zone matrix (glszm) (16), and neighboring gray tone difference matrix (ngtdm) (5). Abbreviations: SUVmax, maximum standardized uptake value; SUVmean, mean of standardized uptake value; MTV, metabolic total volume; TL, total lesion.

Based on SUVmax, SUVmean, MTV, and TL, there were 80, 85, 14, and 28 features from radiomic zone-1 that were able to distinguish responders from non-responders to ADT (Figure 4), respectively. Interestingly, the glcm category included 20 features each for both SUVmax and SUVmean. In addition, the shape category comprised five and nine features that distinguished between different treatment response groups based on MTV and TL, respectively. Based on SUVmax or SUVmean, at least one feature from radiomic zone-1 effectively distinguished responders from non-responders. Importantly, we identified seven features extracted from at least three of the four traditional PET parameters that successfully predicted treatment response (Table 3). Of them, three—including glcm_idmn (*p* = 0.003, 0.002, 0.024, 0.014 for SUVmax, SUVmean, MTV, and TL, respectively), glcm_idn (*p* = 0.003/0.002/0.024/0.014 for SUVmax, SUVmean, MTV, and TL, respectively), and glrlm_rln (*p* = 0.003/0.001/0.013/1.97 × 10^−4^ for SUVmax, SUVmean, MTV, and TL, respectively)—were extracted from all four PET-derived parameters. The remaining four features were glcm_imc1 (*p* = 0.002/7.33 × 10^−41^/0.037 for SUVmax/SUVmean/MTV, respectively), ngtdm_Contrast (*p* = 0.004/0.002/0.037 for SUVmax/SUVmean/MTV, respectively), gldm_dn (*p* = 0.002/0.001/0.009 for SUVmax/SUVmean/TL, respectively), and shape_MeshVolume (*p* = 0.038/0.034/0.003 for SUVmax/SUVmean/TL, respectively).

The glcm_idmn (inverse difference moment normalized) and glcm_idn (inverse difference normalized) features are measures of local homogeneity within ROIs. The glcm_imc1 (informational measure of correlation) feature summarizes the correlation between the probability distributions of texture complexity (complete independency, imc1 = 0; complete dependency, imc1 = −1). The ngtdm_Contrast feature is a measure of spatial intensity changes in each ROI. The glrlm_rln (run length non-uniformity) feature is a measure of the run length similarity throughout an image. Finally, the gldm_dn (dependence non-uniformity) feature expresses the similarity of dependency throughout an image [43].

### 3.3. Prediction of Treatment Response Using Features from Radiomic Zone-2

Based on SUVmax, SUVmean, MTV, and TL, there were 21, 25, 1, and 2 features from radiomic zone-2 that were able to distinguish responders from non-responders to ADT (Figure 4), respectively. The shape category comprised seven, nine, and two features that distinguished between different treatment response groups based on SUVmax, SUVmean, and TL, respectively. We identified only one feature (gldm) that effectively distinguished responders from non-responders based on MTV. Notably, there were two features extracted from at least three of the four traditional PET parameters that successfully predicted treatment response (Table 3). They included gldm_sdlgle (*p* = 0.034/0.027/0.045 for SUVmax, SUVmean, and MTV, respectively) and shape_MinorAxisLength (*p* = 0.018/0.005/0.015 for SUVmax, SUVmean, and TL, respectively). The gldm_sdlgle (small dependence low gray level emphasis) feature is a measure of joint distribution of small dependency with low-intensity SUV for radiomic zone-2. The shape_MinorAxisLength feature expresses the second-largest axis length of each ROI [43].

### 3.4. Prediction of Treatment Response Using Features from Radiomic Zone-3

Based on SUVmax, SUVmean, MTV, and TL, there were three, four, two, and four features from radiomic zone-3 that were able to distinguish responders from non-responders to ADT (Figure 4). The shape feature category comprised one, one, two, and two features that distinguished between different treatment response groups based on SUVmax, SUVmean, MTV, and TL, respectively. Moreover, the first-order, diagnosis, and glcm categories comprised one feature each that distinguished between different treatment response groups. We identified two features extracted from at least three of the four traditional PET parameters that successfully predicted treatment response (Table 3). They included diagnostics_Mask-interpolated_Minimum (*p* = 0.019/0.023/0.038 for SUVmax, SUVmean, and TL, respectively) and shape_Sphericity (*p* = 0.012/0.004/0.034 for SUVmax, SUVmean, and MTV, respectively). The diagnostics_Mask-interpolated_Minimum feature in radiomic zone-3 expresses the minimum SUV measured in the entire prostate gland, with the exclusion of tumor volume. The shape_Sphericity feature is a mathematical quantity that compares the morphology of an object to that of a perfect sphere. The shape_Sphericity of a perfect sphere is equal to one.

### 3.5. Surface Volume Ratio in the Three Radiomic Zones

On analyzing the features that distinguished responders from non-responders to ADT, we found that shape_SurfaceVolumeRatio (SVR) was simultaneously present in all of the three radiomic zones. Specifically, SVR successfully predicted treatment response according to RR changes based on MTV (*p* = 0.017) and TL (*p* = 3.49 × 10^−4^) in radiomic zone-1; SUVmax (*p* = 0.017) and SUVmean (*p* = 0.018) in radiomic zone-2; and SUVmax (*p* = 0.01) and MTV (*p* = 0.027) in radiomic zone-3, respectively (Table 3).

## 4. Discussion

**Figure 5:**
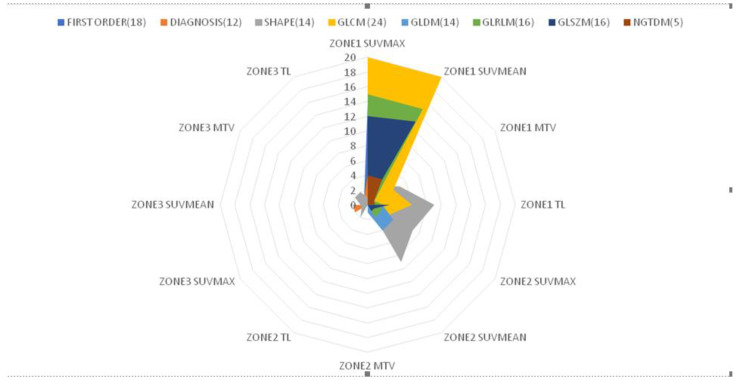
Radar chart depicting the correlations between different features extracted from the three radiomic zones and response to androgen deprivation therapy in patients with prostate cancer.

Prediction of response to ADT prior to treatment initiation is a difficult task. In this study, we were able to identify several features from prostate radiomic zones that were able to successfully predict response to ADT in patients with PCa. As expected, the highest number of predictive features was identified within radiomic zone-1 (i.e., the metabolic tumor zone; Figure 5). Specifically, seven features extracted from at least three of the four traditional PET parameters were significantly associated with ADT outcomes. Responders to ADT were more likely to have lower glcm_idmn, glcm_idn, glcm_imc1, glrlm_rln, gldm_dn, and shape_MeshVolume values as well as higher ngtdm_Contrast values (Table 4). Of note, five of these features (i.e., glcm_idmn, glcm_idn, glcm_imc1, ngtdm_Contrast, and glrlm_rln) were associated with the texture distribution properties of PET images [43] which are in turn strongly correlated with intra- and inter-tumor heterogeneity [44,45,46] and treatment response [26].

A strength of this study is that the analysis of predictive radiomic features was not limited to the main metabolic tumor zone (i.e., zone-1). Accordingly, certain features from both zone-2 and zone-3 were also able to distinguish between treatment response groups. Findings from zone-2 suggested that responders to ADT were more likely to have lower shape_MinoAxisLength and higher gldm_sdlgle values, whereas data from zone-3 revealed that the ADT response group had lower diagnostics_Mask-interpolated_Minimum and higher shape_Sphericity values (Table 4). These results indicate that radiomic characteristics extracted from peripheral prostatic zones may also have value in the prediction of ADT response. We have previously shown that distinct radiomic zones are useful for classifying patients with PCa in different risk groups [35]. In another study, Rodrigues et al. [47] demonstrated that features extracted from tumor-surrounding regions are strongly associated with Gleason scores. By taking zone-2 and zone-3 into account, we extracted as much radiomics information as possible to assist prediction of ADT treatment outcomes during the pretreatment phase.

Within radiomic zone-2, two features (gldm_sdlgle and shape_MinorAxisLength) successfully predicted treatment response. This observation suggests that joint distribution of small dependency with low-intensity SUV (gldm_sdlgle) and the second-largest axis length (shape_MinorAxisLength) of each ROI in this zone are associated with treatment outcomes. This could reflect the paramount role played by the ring region surrounding the primary tumor volume in limiting cancer spread to both lymph nodes and distant sites. In general, radiomic zone-2 was characterized by lower SUV values and less heterogeneity compared to zone-1. Two features from radiomic zone-3 (diagnostics_Mask-interpolated_Minimum and shape_Sphericity) were also significantly associated with ADT outcomes. Previously, the same features extracted from ^11^C-choline PET images successfully differentiated between high- and low-risk PCa [30]. An interesting observation from our study is that SVR was the only feature identified as being associated with response to ADT in all of the three radiomic zones. Specifically, responders to ADT were more likely to show higher SVR values from both zone-1 and zone-2 and lower SVR values from zone-3 (Table 4). Notably, Cuocolo et al. [48] have recently demonstrated that SVR was the strongest independent predictor of clinically significant PCa among all of the MRI shape features taken into account.

There are several limitations to our study. First, its single-center design may have limited the external validity of the results. Second, only 35 patients were included. A larger sample size might have improved the power of the study in terms of identifying between-group differences and, for that reason, larger prospective cohorts are needed. A longer follow-up is also necessary to confirm our findings and to evaluate whether the radiomic features identified in our study are correlated with clinical response to ADT.

## 5. Conclusions

Seven features extracted from radiomic zone-1 were significantly associated with ADT outcomes in patients with PCa. Two features from zone-2 and two from zone-3 were also able to distinguish between different treatment response groups. If independently validated in larger studies, feature analysis of different radiomic zones within the prostate gland could be useful to differentiate responders from non-responders before the initiation of ADT.

## Figures and Tables

**Table 1:** General characteristics of the 35 study patients with advanced prostate cancer.

Characteristic	Value, *n* (%)
Age, years (mean ± SD)	70 ± 9.9
Stage (AJCC Manual, eighth edition)	
IIIB	7 (20%)
IIIC	3 (9%)
IVA	8 (23%)
IVB	17 (48%)
Serum prostate-specific antigen (ng/mL)	
<10	4 (11%)
10–20	9 (26%)
>20	22 (63%)
Gleason score	
7	11 (31%)
8	5 (14%)
9	16 (46%)
10	3 (9%)
ISUP grade	
2	3 (9%)
3	7 (20%)
4	5 (14%)
5	20 (57%)
ADT regimen	
Leuprorelin + bicalutamide	10 (28%)
Leuprorelin + cyproterone	4 (11%)
Leuprorelin + abiraterone	2 (6%)
Triptorelin + cyproterone	1 (3%)
Goserelin + bicalutamide	9 (26%)
Leuprorelin + abiraterone + bicalutamide	2 (6%)
Leuprorelin	2 (6%)
Goserelin	4 (11%)
Degarelix	1 (3%)

Data are given as counts and percentages in parentheses, unless otherwise indicated. Abbreviations: SD, standard deviation; AJCC, American Joint Committee on Cancer; ISUP, International Society of Urological Pathology; ADT, androgen deprivation therapy.

**Table 2:** Response to androgen deprivation therapy treatment: patient-based classification groups.

PET Parameter and Classification		Prostate Tumor *n* = 35 (%)	Metastatic Nodes *n* = 16 (%)	Bone Metastases *n* = 17 (%)
SUVmax	Response	27 (77%)	13 (81%)	13 (76%)
	No response	8 (23%)	3 (19%)	4 (24%)
SUVmean	Response	26 (74%)	14 (87%)	10 (59%)
	No response	9 (26%)	2 (13%)	7 (41%)
MTV	Response	21 (60%)	14 (87%)	13 (76%)
	No response	14 (40%)	2 (13%)	4 (24%)
TL	Response	29 (83%)	15 (94%)	14 (82%)
	No response	6 (17%)	1 (6%)	3 (18%)

Patients in the response group were those who showed a reduction ratio > 30% for PET-derived parameters measured on PET-2 versus PET-1. All other patients were included in the no response group. Abbreviations: SUV, standardized uptake value; MTV, metabolic total volume; TL, total lesion.

**Table 3:** ^68^Ga-PSMA-11 PET/CT features extracted from different radiomic zones in the prediction of response to androgen deprivation therapy in patients with advanced prostate cancer.

Category	Feature	Zone-1				Zone-2				Zone-3			
		SUVmax	SUVmean	MTV	TL	SUVmax	SUVmean	MTV	TL	SUVmax	SUVmean	MTV	TL
glcm	idmn	0.010	0.005	0.018	0.004	0.024	0.008	_	_	_	_	_	_
	idn	0.003	0.002	0.024	0.014	0.023	0.009	_	_	_	_	_	_
	imc1	0.002	7.33 × 10^−41^	0.037	_	_	_	_	_	_	_	_	_
ngtdm	Contrast	0.004	0.002	0.037	_	_	0.034	_	_	_	_	_	_
glrlm	rln	0.003	0.001	0.013	1.97 × 10^−4^	0.031	0.012	_	_	_	_	_	_
gldm	dn	0.002	0.001	_	0.009	_	_	_	_	_	_	_	_
Shape	MeshVolume	0.038	0.034	_	0.003	0.01	0.005	_	_	_	_	_	_
gldm	sdlgle	_	_	_	_	0.034	0.027	0.045	_	_	_	_	_
shape	MinorAxisLength	_	_	0.050	0.025	0.018	0.005	_	0.015	_	_	_	_
	Sphericity	_	_	_	_	_	_	_	_	0.012	0.004	0.034	_
diagnosis	Mask-interpolated_Minimum	0.034	0.025	_	_	_	_	_	_	0.019	0.023	_	0.038
shape	SurfaceVolumeRatio	_	_	0.017	3.49 × 10^−4^	0.017	0.018	_	_	0.010	_	0.027	_

Abbreviations: SUV, standardized uptake value; MTV, metabolic total volume; TL, total lesion; glcm, gray level co-occurrence matrix; idmn, inverse difference moment normalized; idn, inverse difference normalized; imc1, informational measure of correlation 1; ngtdm, neighboring gray tone difference matrix; glrlm, gray level run length matrix; rln, run length non-uniformity; gldm, gray level dependence matrix; dn, dependence non-uniformity; sdlgle, small dependence low gray level emphasis. Significant *p* values are shown in the table.

**Table 4:** Medians and interquartile ranges (IQRs) of predictive features identified within radiomic zone-1, zone-2, and zone-3 in responders and non-responders to androgen deprivation therapy.

Feature	Responders/Non-Responders (Median ± IQR)	
**Zone-1**	**SUVmax**	**SUVmean**
glcm_idmn	0.948 ± 0.036/0.970 ± 0.022	0.947 ± 0.036/0.968 ± 0.019
glcm_idn	0.850 ± 0.054/0.890 ± 0.043	0.850 ± 0.053/0.885 ± 0.037
glcm_imc1	−0.480 ± 0.213/−0.232 ± 0.168	−0.473 ± 0.341/−0.196 ± 0.096
ngtdm_Contrast	0.853 ± 1.892/0.153 ± 0.494	0.853 ± 2.144/0.210 ± 0.477
glrlm_rln	211.2 ± 247.7/362.1 ± 1019.3	206.9 ± 226.4/528.9 ± 908.7
gldm_dn	114.0 ± 104.5/196.3 ± 81.12	93.42 ± 98.73/97.44 ± 214.4
shape_MeshVolume	4616 ± 6799/9411 ± 23988	4460 ± 5463/13,303 ± 20721
shape_SurfaceVolumeRatio	0.451 ± 0.159/0.396 ± 0.262	0.451 ± 0.155/0.354 ± 0.241
**Zone-2**		
gldm_sdlgle	0.034 ± 0.0225/0.020 ± 0.0165	0.034 ± 0.024/0.0216 ± 0.015
shape_MinoAxisLength	39.18 ± 12.64/46.90 ± 6.837	38.43 ± 12.71/47.00 ± 7.060
shape_SurfaceVolumeRatio	0.558 ± 0.218/0.368 ± 0.108	0.509 ± 0.279/0.426 ± 0.191
**Zone-3**		
shape_Sphericity	0.609 ± 0.093/0.525 ± 0.186	0.614 ± 0.091/0.497 ± 0.152
diagnostics_Mask-interpolated_Minimum	0.204 ± 0.112/0.320 ± 0.163	0.199 ± 0.127/0.299 ± 0.150
shape_SurfaceVolumeRatio	0.173 ± 0.052/0.211 ± 0.081	0.175 ± 0.053/0.197 ± 0.079

Abbreviations: SUV, standardized uptake value; glcm, gray level co-occurrence matrix; idmn, inverse difference moment normalized; idn, inverse difference normalized; imc1, informational measure of correlation 1; ngtdm, neighboring gray tone difference matrix; glrlm, gray level run length matrix; rln, run length non-uniformity; gldm, gray level dependence matrix; dn, dependence non-uniformity; sdlgle, small dependence low gray level emphasis.

## Data Availability

The datasets generated and/or analyzed during the current study are available from the corresponding author on reasonable request.
